# Inflammatory bowel disease and prostate cancer risk: a two-sample Mendelian randomization analysis

**DOI:** 10.3389/fimmu.2023.1157313

**Published:** 2023-06-20

**Authors:** Wen Cheng, Yang Liao, Ruiyu Mou, Xian Xiao, Yingjie Jia

**Affiliations:** ^1^Department of Oncology, First Teaching Hospital of Tianjin University of Traditional Chinese Medicine, Tianjin, China; ^2^National Clinical Research Center for Chinese Medicine Acupuncture and Moxibustion, Tianjin, China

**Keywords:** inflammatory bowel disease, prostate cancer, Mendelian randomization, causality, Crohn ‘s disease, ulcerative colitis

## Abstract

**Background:**

Previous epidemiological observational studies have reported an association between inflammatory bowel disease (IBD) and prostate cancer (PCa), but the causality is inconclusive. The purpose of this study was to evaluate the causality of IBD on PCa using the mendelian randomization (MR) analysis.

**Methods:**

We performed a two-sample MR analysis with public genome-wide association studies (GWAS) data. Eligible instrumental variables (IVs) were selected according to the three assumptions of MR analysis. The inverse-variance weighted (IVW) method was the main method. Complementary methods included the MR-Egger regression, the Weighted Median, the Simple Mode, the Weighted Mode and MR pleiotropy residual sum and outlier (MR-PRESSO) methods.

**Results:**

Genetically determined IBD did not have a causal effect on PCa (IVW *P* > 0.05). Additionally, there was no causal effect of Crohn’s disease (CD) and ulcerative colitis (UC) on PCa in the MR analysis (IVW *P* > 0.05). Results of complementary methods were consistent with those of the IVW method.

**Conclusions:**

This study does not support a causal association of IBD on PCa, which is in contrast to most observational studies.

## 1 Introduction

Prostate cancer (PCa) is the second most common cancer in men and represents one of the main causes of cancer-related death worldwide (1). Despite recent advances in PCa treatment, its incidence varies greatly in different regions of the world, but all show a steady increase annually (2). The most well-established risk factors are age, family history and ethnic background, while other aetiological factors remain controversial (3). Recently, several exogenous factors have been discussed to be associated with the risk of PCa, such as obesity, diabetes, metabolic syndrome and dietary factors (4, 5). Given the large burden of PCa globally, modifiable risk factors that could lower the incidence of PCa must be identified.

Inflammatory bowel disease (IBD), comprising Crohn’s disease (CD) and ulcerative colitis (UC), is a common chronic relapsing idiopathic disease (6). It is reported that the incidence and prevalence of IBD has risen over the past decade to become a global public health problem (7). Inflammation is closely associated with tumorigenesis and cancer progression. Mounting evidence suggests that patients with IBD are at increased risk of gastrointestinal and extraintestinal malignancies, including colorectal cancer and melanoma (8, 9). In light of the anatomical proximity of the gastrointestinal tract to the prostate, several epidemiological studies have evaluated the association between IBD and PCa risk, but the results are inconsistent. Several cohort studies demonstrated that men with IBD had an increased PCa risk compared to men without IBD (10, 11). Results from a meta-analysis implied that the prevalence of PCa in IBD patients, especially UC, was higher than in healthy controls (12). Wilson et al. reported that there was no increased PCa risk overall in individuals with IBD compared to IBD-free individuals (13). On account of potential confounding and reverse causation, traditional observational studies are hard to provide conclusive evidence. Therefore, the causal role of IBD in the occurrence of PCa remains unclear.

Mendelian randomization (MR) analysis is an effective approach for investigating the causal relationship between two traits, which may overcome the limitations of traditional observational studies to some extent (14). Using genetic variants as instrumental variables (IVs), MR provides a natural RCT and enriches epidemiological research methods. With the increasing size and scope of genome-wide association studies (GWAS), two-sample MR analysis has been widely used in a variety of diseases. Here, we applied a two-sample MR analysis to evaluate the causality of IBD on PCa based on summary statistics from large scale GWAS.

## 2 Methods

**Figure 1:**
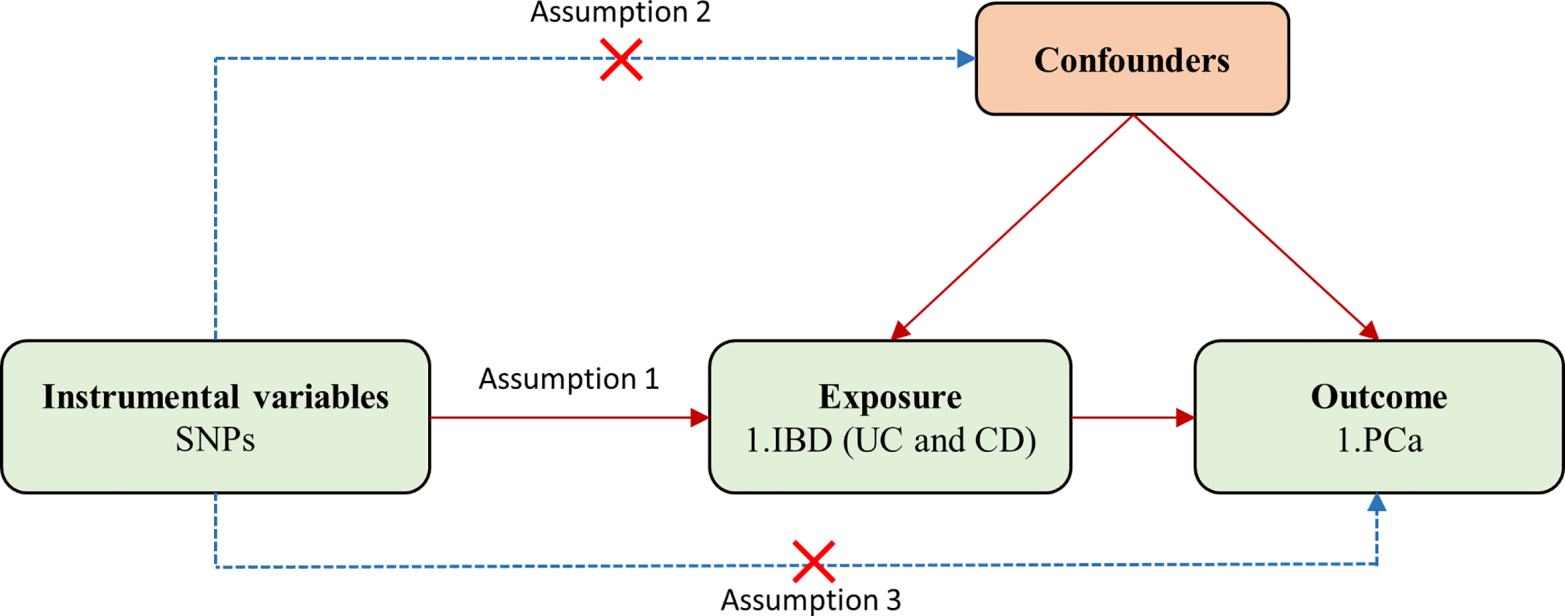
Flowchart for the two-sample Mendelian randomization analysis. SNPs, single-nucleotide polymorphisms; PCa, prostate cancer; IBD, inflammatory bowel disease; UC, ulcerative colitis; CD, Crohn’s disease.

In the two-sample MR analysis, single-nucleotide polymorphisms (SNPs) used as IVs should satisfy three key assumptions (**Figure 1**): (1) IVs are strongly associated with exposure; (2) IVs are independent of any confounding factors; (3) IVs affect the outcome only through the exposure.

### 2.1 GWAS summary data for IBD and PCa

In order to obtain a more reliable conclusion of the causal relationship, we searched eligible summary-level data from the largest public GWAS for each trait (**Table 1**). Since all data used was published previously in the public database, no additional ethical approval was needed. Specifically, summary statistics for PCa and IBD (including UC and CD) were extracted from the IEU OpenGWAS project (https://gwas.mrcieu.ac.uk/datasets/). Diagnostic criteria and inclusion procedures refer to the original literature. Besides, all participants in the study were of European ancestry without sample overlap between the exposure and outcome traits, which minimize the bias caused by confounding factors.

**Table 1:** Characteristics of PCa and IBD GWAS cohorts.

Disease	Study	Journal	Cases	Controls	Sample size	GWAS ID
IBD	M. de Lange et al. (15)	Nat Genet.	25,042	34,915	59,957	ebi-a-GCST004131
CD	M. de Lange et al. (15)	Nat Genet.	12,194	28,072	40,266	ebi-a-GCST004133
UC	M. de Lange et al. (15)	Nat Genet.	12,366	33,609	45,975	ebi-a-GCST004132
PCa	Schumacher FR et al.1 (16)	Nat Genet.	79,148	61,106	140,254	ieu-b-85

IBD, inflammatory bowel disease; CD, Crohn’s disease; UC, ulcerative colitis; PCa, prostate cancer.

IBD, inflammatory bowel disease; CD, Crohn’s disease; UC, ulcerative colitis; PCa, prostate cancer

### 2.2 Selection of genetic instrumental variables

Based on the above GWAS summary data, the strict process was performed to identify eligible SNPs as IVs. Firstly, SNPs should be strongly related to exposure with a genome-wide significance level of *P*-value < 5×10^-8^. Secondly, to avoid biased results caused by linkage disequilibrium (LD), we conducted the clumping process with a cutoff of *R*^2 = ^0.001 and window size = 10,000 kb. Thirdly, the Phenoscanner database (http://www.phenoscanner.medschl.cam.ac.uk/) was used to screen genetic variants which were associated with confounding factors. Fourthly, if the above screened SNPs were not available in the outcome GWAS data, proxy SNPs (high LD of *R*^2^ >0.8 with the target SNPs) would be searched as substitutes. Finally, the exposure and outcome datasets were harmonized to restrict palindromic and ambiguous SNPs with non-concordant alleles, thus keeping the effect alleles on the exposure and outcome uniform.

Besides, in order to better satisfy key assumption one, we calculated the *F* statistic for each SNP solely. Weak IVs tend to be *F* statistics < 10 and would be removed in further analyses (17). After above filtering steps, these carefully selected SNPs were used as final IVs for subsequent two-sample MR analysis.

### 2.3 Statistical analysis

In our study, multiple methods were applied to estimate the causal associations and effect between exposure and outcome. The inverse variance weighted (IVW), the most widely used method in MR analysis, had the fixed-effects and the random-effects versions. As a meta-analysis approach, IVW obtained the total estimates of the effect of exposure on outcome by combining Wald estimates of causality for each IV (18). Several complementary approaches were established to provide robust causal estimates under different assumptions, including the MR-Egger regression, the Weighted Median, the Simple Mode, the Weighted Mode and MR pleiotropy residual sum and outlier (MR-PRESSO) methods. Based on the assumption of Instrument Strength Independent of Direct Effect (InSIDE), the MR-Egger regression method performed a weighted linear regression to produce a consistent causal effect estimate independent of IV validity (19, 20). Furthermore, the intercept term of MR-Egger regression method was considered as an indicator of directional pleiotropic effects, and *P*-value < 0.05 was considered to have pleiotropy. Nevertheless, the MR-Egger regression method was relatively poor in accuracy and susceptible to outlying genetic variants. The Weighted Median method was able to achieve unbiased estimates of effects, which did not demand the InSIDE assumption and thus presenting remarkable advantages over the MR-Egger regression method. Concretely, it was an outstanding auxiliary method providing lower type I error by examining the weighted median value of the ratio instrumental variable estimates (21). At last, the Weighted Mode method was used to assess the overall causal effect from a large number of genetic instruments. In many situations, this method produced lower type-I error rates, less bias and smaller power than main methods (22).

In addition to MR-Egger regression method, MR-PRESSO method was performed to test and process pleiotropy (23). It was carried out according the following procedures: (1) evaluation of horizontal pleiotropy; (2) correction for horizontal pleiotropic outliers; (3) assessing whether the causal effect was significantly different (*P* < 0.05) after outlier removal. The number of distributions in MR-PRESSO analysis was set to 1000. Cochran’s Q statistic was calculated to quantify the heterogeneities detected by the IVW and MR-Egger regression methods, and a *P*-value less than 0.05 was considered heterogeneous and thus a random-effect model was applied for subsequent analyses. Otherwise, a fixed-effect model was used (24). Besides, the “leave-one-out” sensitivity analysis was applied for exploring whether there was a single SNP which created bias to influence the overall causal effect. The statistical power was calculated by the mRnd website (https://shiny.cnsgenomics.com/mRnd/) (25).

The two-sample MR analysis was conducted using packages “TwoSampleMR” (26) and “MRPRESSO” in open-source statistical software R (version 4.1.2, R Foundation for Statistical Computing, Vienna, Austria).

## 3 Results

### 3.1 Causal effects of IBD on PCa

After LD clumping, proxy SNP exploration, the Phenoscanner database mining and data harmonization, we selected eligible SNPs as IVs to fit three key assumptions. The number of SNPs were 103 for IBD, 82 for CD and 53 for UC. The *F* statistic for every variant was much > 10 in our study. Average *F* statistic values were 70.93, 79.54, and 69.85 for IBD, CD and UC, presenting the small possibility of weak instrumental variable bias. The results of power analysis were shown in **Table 2**, all statistical power rates were greater than 80%, indicating high credibility. Detailed information of IVs for IBD and subtypes was listed in **Supplementary Table S1-S6**.

**Table 2:** Power calculation for all the MR analysis in current study.

Exposure		Outcome		SNPs number	Proportion of variance explained by the SNPs onexposure	Power (%)
Trait	Sample size	Trait	Sample size			
IBD	59,957	PCa	140,254	103	0.41	95
CD	40,266	PCa	140,254	82	0.50	98
UC	45,975	PCa	140,254	53	0.57	81

IBD, inflammatory bowel disease; CD, Crohn’s disease; UC, ulcerative colitis; PCa, prostate cancer.

IBD, inflammatory bowel disease; CD, Crohn’s disease; UC, ulcerative colitis; PCa, prostate cancer

**Figure 2:**
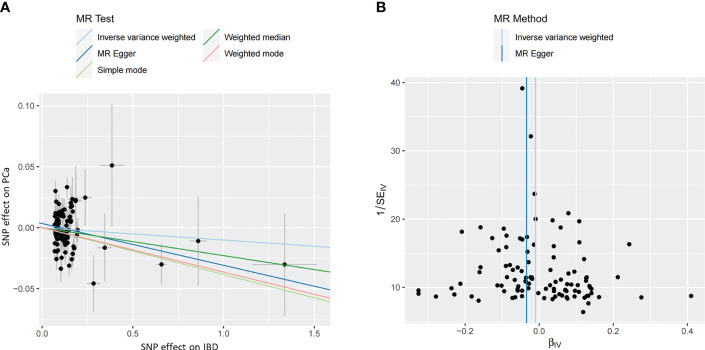
**(A)** The scatter plot of the causal effect of IBD on PCa risk. Analyses were conducted using the inverse-variance weighted, MR-Egger, Weighted Median, Simple Mode, and Weighted Mode methods. The slope of each line corresponding to the causal estimates for each method. **(B)** The funnel plot of the causal effect of IBD on PCa risk. Individual SNP was delineated in the background. PCa, prostate cancer; IBD, inflammatory bowel disease; SNP, single nucleotide polymorphism.

The MR estimates from different approaches of evaluating the causal effect of IBD and subtypes on PCa were presented in **Table 3**. The results of sensitivity analysis were presented in **Table 4**. The Cochran’s Q test showed significant heterogeneity (*P* < 0.001), so we applied the IVW method with the random-effect model. The results of IVW analysis found that IBD had no causal effect on PCa (OR = 0.97, 95% CI: 0.94-1.02, *P* = 0.326), and the MR-Egger, the Weighted Median, and the Simple Mode methods showed consistent results. Only the Weighted mode method found a significant causal effect of IBD on PCa (OR = 0.96, 95% CI: 0.93-0.99, *P* = 0.049). No directional pleiotropy was identified by MR-Egger regression method (intercept = 0.003, *P* = 0.232). The MR-PRESSO method detected horizontal pleiotropy (*P* < 0.001) and three outliers (rs11236797, rs5763793, rs6062496). However, after removing outliers, results of MR analysis showed no significant change (IVW OR=0.99, 95% CI: 0.97-1.01, *P* = 0.197) and the heterogeneity still existed (*P* < 0.001). The scatter plot and funnel plot were shown in **Figure 2**. The results of leave-one-out sensitivity and single SNP risk analysis were shown in **Supplementary Figure S1** and **Supplementary Table S7**.

**Table 3:** MR analysis of the causality of IBD on PCa.

Exposure	Outcome	Number of SNPs	*F* statistic	Methods	β (95%CI)	OR (95%CI)	SE	*P*
IBD	PCa	103	70.93	MR Egger	-0.03 (-0.08, 0.01)	0.97 (0.92, 1.01)	0.02	0.132
				Weighted median	-0.02 (-0.05, 0.01)	0.98 (0.95, 1.01)	0.01	0.065
				IVW	-0.03 (-0.06, 0.02)	0.97 (0.94, 1.02)	0.01	0.326
				Simple mode	-0.04 (-0.10, 0.03)	0.96 (0.90, 1.03)	0.03	0.245
				Weighted mode	-0.04 (-0.07, -0.01)	0.96 (0.93, 0.99)	0.01	0.049
CD	PCa	82	79.54	MR Egger	-0.02 (-0.06, 0.03)	0.98 (0.94, 1.03)	0.02	0.427
				Weighted median	-0.01 (-0.03, 0.01)	0.99 (0.97, 1.01)	0.01	0.372
				IVW	-0.03 (-0.07, 0.01)	0.97 (0.93, 1.01)	0.01	0.235
				Simple mode	-0.04 (-0.09, 0.01)	0.96 (0.92, 1.01)	0.02	0.093
				Weighted mode	-0.01 (-0.04, 0.02)	0.99 (0.96, 1.02)	0.01	0.496
UC	PCa	53	69.85	MR Egger	-0.06 (-0.13, 0.01)	0.94 (0.88, 1.01)	0.03	0.078
				Weighted median	-0.03 (-0.05, 0.01)	0.97 (0.95, 1.01)	0.01	0.052
				IVW	-0.02 (-0.04, 0.02)	0.98 (0.96, 1.02)	0.01	0.416
				Simple mode	-0.03 (-0.10, 0.03)	0.97 (0.91, 1.03)	0.03	0.334
				Weighted mode	-0.04 (-0.08, -0.01)	0.96 (0.93, 0.99)	0.02	0.043

IBD, inflammatory bowel disease; CD, Crohn’s disease; UC, ulcerative colitis; PCa, prostate cancer; IVW, inverse variance weighted; OR, odds ratio; CI, confidence interval; SE, standard error.

**Table 4:** Sensitivity analyses of MR.

Exposure	Outcome	Horizontal pleiotropy						Heterogeneity					
		MR-PRESSO global outlier test				MR-Egger regression		MR-Egger			IVW		
		*P*	Outlier	OR (95% CI)*	P*	Intercept	*P*	Q statistic	*P*	*P*^#^	Q statistic	*P*	*P*^#^
IBD	PCa	<0.001	rs11236797, rs5763793, rs6062496	0.99(0.97,1.01)	0.197	0.003	0.232	190.99	<0.001	<0.001	193.72	<0.001	<0.001
CD	PCa	<0.001	rs11236797, rs6062496, rs6808936	0.99(0.97,1.01)	0.085	0.001	0.711	157.45	<0.001	0.063	157.72	<0.001	0.067
UC	PCa	<0.001	rs2212434, rs6062496	0.99(0.97,1.01)	0.318	0.009	0.112	104.79	<0.001	0.011	110.15	<0.001	0.004

* MR analysis using IVW method after removing outliers identified by MR-PRESSO method.

# Heterogeneity test after removing outliers.

IBD, inflammatory bowel disease; CD, Crohn’s disease; UC, ulcerative colitis; PCa, prostate cancer; IVW, inverse variance weighted; OR, odds ratio; CI, confidence interval; SE, standard error.

### 3.2 Causal effects of CD on PCa

In addition, the results of MR analysis demonstrated that genetically predicted CD was not associated with PCa (OR = 0.97, 95% CI: 0.93-1.01, *P* = 0.235). The MR-Egger regression method did not identify horizontal pleiotropy (intercept = 0.001, *P* = 0.711). Using the MR-PRESSO method, we found horizontal pleiotropy (*P* < 0.001) and three SNPs (rs11236797, rs6062496, rs6808936) were identified as outliers. Results of MR analysis did not change after excluding abnormal SNPs (IVW OR=0.99, 95% CI: 0.97-1.01, *P* = 0.085), but the heterogeneity was disappeared (MR-Egger *P* = 0.063, IVW *P* = 0.067). **Supplementary Figure S2** exhibited the visualization results of causal effects of CD on PCa. **Supplementary Table S8** presented the results of leave-one-out sensitivity and single SNP risk analysis.

### 3.3 Causal effects of UC on PCa

There was no causal relationship between UC and PCa based on IVW approach (OR=0.98, 95% CI: 0.96-1.02, *P* = 0.416). The Weighted mode method found UC was associated with PCa (OR = 0.96, 95% CI: 0.93-0.99, *P* = 0.043). Horizontal pleiotropy between IVs and outcome was not observed by the MR-Egger regression method (intercept = 0.009, *P* = 0.112). The MR-PRESSO method showed the existence of heterogeneity (*P* < 0.001) and two outliers (rs2212434, rs6062496). After removing outliers, the results did not change (IVW OR=0.99, 95% CI: 0.97-1.01, *P* = 0.318), and the heterogeneity still existed (MR-Egger *P* = 0.011, IVW *P* = 0.004). The visualization results of causal relationships of UC on PCa were provided in **Supplementary Figure S3**. The results of leave-one-out sensitivity and single SNP risk analysis were presented in **Supplementary Table S9**.

## 4 Discussion

This was the first study to comprehensively examine the causal effect of IBD (including UC and CD) on PCa with summary GWAS data. The overall results of our two-sample MR analysis failed to find any evidence that genetically predicted IBD and subtypes were causally linked to the risk of PCa.

In recent years, several epidemiological studies have noted the link between IBD and PCa, which was contrary to our findings. A retrospective, matched-cohort study including 1,033 cases and 9,306 healthy controls concluded that men with IBD had increased risk of clinically significant PCa incidence at 10 years (HR = 4.04, 95% CI: 2.52-6.48, *P* < 0.001) (10). Besides, Mosher et al. found IBD patients had a significantly higher risk for prostate cancer (HR = 1.70, 95% CI: 1.28-2.27) (27). Although these studies matched age and ethnicity to minimize confounding effects, it was difficult to avoid the inherent limitations of retrospective studies. Therefore, another population-based study involving 218,084 men assessed the association between IBD and subsequent PCa from a prospective perspective, and reported an increased risk of PCa for men with IBD (HR = 1.31, 95% CI: 1.03-1.67, *P* = 0.029). According to subtypes, the relation with PCa was only among men with UC (HR = 1.47, 95% CI: 1.11-1.95, *P* = 0.007), and not CD (HR = 1.06, 95% CI: 0.63-1.80, *P* = 0.820) (11). In addition, a meta-analysis of nine observational studies (six cohort and three case-control studies), including 409,748 participants, demonstrated that men with IBD especial UC had significantly elevated PCa risk (12). The real-world data above have suggested that men with IBD may have a higher susceptibility to PCa, but a nationwide register study in Finland found that males with UC had a slightly decreased risk of PCa (SIR = 0.79, 95% CI: 0.52-1.16) (28).

However, not all observational studies proved the existence of a direct causal relationship between IBD or subtypes and PCa. A nested case–control analysis based on the data from the clinical practice research datalink (CPRD) revealed that there was no association between IBD and PCa (13). Tim et al. studied the risk of PCa in the Dutch population-based IBDSL cohort including 1,157 CD and 1,644 UC patients, and found no evidence of an increased prevalence of PCa in diagnosed IBD patients compared to healthy controls (CD SIR = 0.23, 95% CI: 0.01-1.26; UC SIR = 1.37, 95% CI: 0.89-2.02) (29).

Given that association studies couldn’t answer causality questions, so it was hard to confirm the causality of IBD on PCa based purely on observational studies. In summary, above findings should be interpreted with caution. In disagreement with the most observational studies, we discovered no causal link of IBD on PCa in our investigation. Obviously, the different analytic approaches could account for differing findings between these researches and our study. Observational studies might be particularly susceptible to confounding factors, while MR analysis could avoid the weakness to produce a relatively accurate causal judgment.

There are a couple of possible reasons that may contribute to the association between IBD and PCa in observational studies. First, inflammatory effect could play a vital role in the occurrence of PCa. IBD-associated local inflammation frequently involves the rectum, and then the adjacent anatomical location facilitates the spread of inflammation to the prostate (10). It is well known that chronic prostate inflammation may be a suspected risk factor for PCa, and the mechanisms contains oxidative stress and epigenetic alterations (30). Furthermore, IBD results in a systemic inflammatory response with higher serum acute phase reactants (31). Studies have demonstrated that higher serum acute phase reactants were correlated with elevated PSA values (32). Various previous researches have confirmed that modification of the intestinal microflora was a critical factor in the process of IBD. Similarly, emerging evidence indicated that gut microbiota disorders, especially harmful bacteria derived from IBD, could induce cancer-promoting prostatic inflammation (33). Therefore, it may be reasonable to infer that the inflammatory effect from IBD induce prostatitis, leading to PCa. However, these speculations await confirmation by further research. Second, immunosuppressive therapy may partly explain the link between IBD and PCa. The major IBD management strategy involves the use of immunosuppressive agents to mitigate the autoimmune activity and control symptoms. Evidence has been shown that immunosuppressive therapy for IBD seemed to be associated with a higher risk of developing extraintestinal cancer. According to the report by Jessica, IBD patients exposed to immunosuppressive therapy tended to develop medium-high risk cervical abnormalities/cancer compared to IBD patients who lacked immunosuppressive therapy (34). Another study performed a medication analysis on IBD and observed that immunosuppression exposure more than one year was related to an increased risk of overall cancer, such as hematologic cancer and squamous cell carcinoma (29). Nevertheless, there is no direct evidence to exactly elucidate correlations between these immunosuppressive drugs and PCa risk among patients with IBD. Future studies could focus more on immunosuppressive drug use regimens, including duration and dosage.

There are several advantages in our MR study. First, to the best of our knowledge, this is the first study to evaluate the causality of IBD on PCa based on a two-sample MR analysis with large scale GWAS data. Compared to previous observational studies, MR analysis could effectively reduce potential bias including confounders and reverse causation, thus enhancing the causal inference. Second, GWAS datasets of IBD and PCa applied were predominately based on populations of European ancestry, which was capable to minimize the impact of population stratification. Third, different estimation models and rigorous sensitivity analysis were used to ensure the reliability and robustness of the results. Meanwhile, we would like to acknowledge some limitations. First, the study included a single population, and the representativeness of the results remains to be further verified in the whole population. Second, although a series of strict steps were used to identify outlier variants for avoiding horizontal pleiotropy, we still unable to totally eliminate the impact of horizontal pleiotropy, which may be due to the complex and unclear biological function of many genetic variants. Third, larger sample sizes and more advanced methods are needed to corroborate the results and fully illustrate the statistical power. Finally, GWAS could provide new insights into genes involved in PCa, but the precise mechanisms studies are needed for better understanding the pathophysiology.

In conclusion, our MR analysis reveals no causal effect of genetically predicted IBD and subtypes on PCa, which is in contrast to most observational studies. To verify the accuracy of our results, future researches based on higher quality GWAS data and more advanced methods are required.

## Data availability statement

The original contributions presented in the study are included in the article/**Supplementary Material**. Further inquiries can be directed to the corresponding author.

## Author contributions

WC and YL contributed equally to this work. YJ constructed this study. Other authors offer advice. All authors contributed to the article and approved the submitted version.
