# Supplementary material for: Inflammatory bowel disease and prostate cancer risk: a two-sample Mendelian randomization analysis
Source: Front Immunol. 2023 Jun 20;14:1157313. doi: 10.3389/fimmu.2023.1157313 (PMC10318899; doi:10.3389/fimmu.2023.1157313)
Supplement: Supplementary file 1 [file DataSheet_1.docx]

**Supplementary Figure S1**.
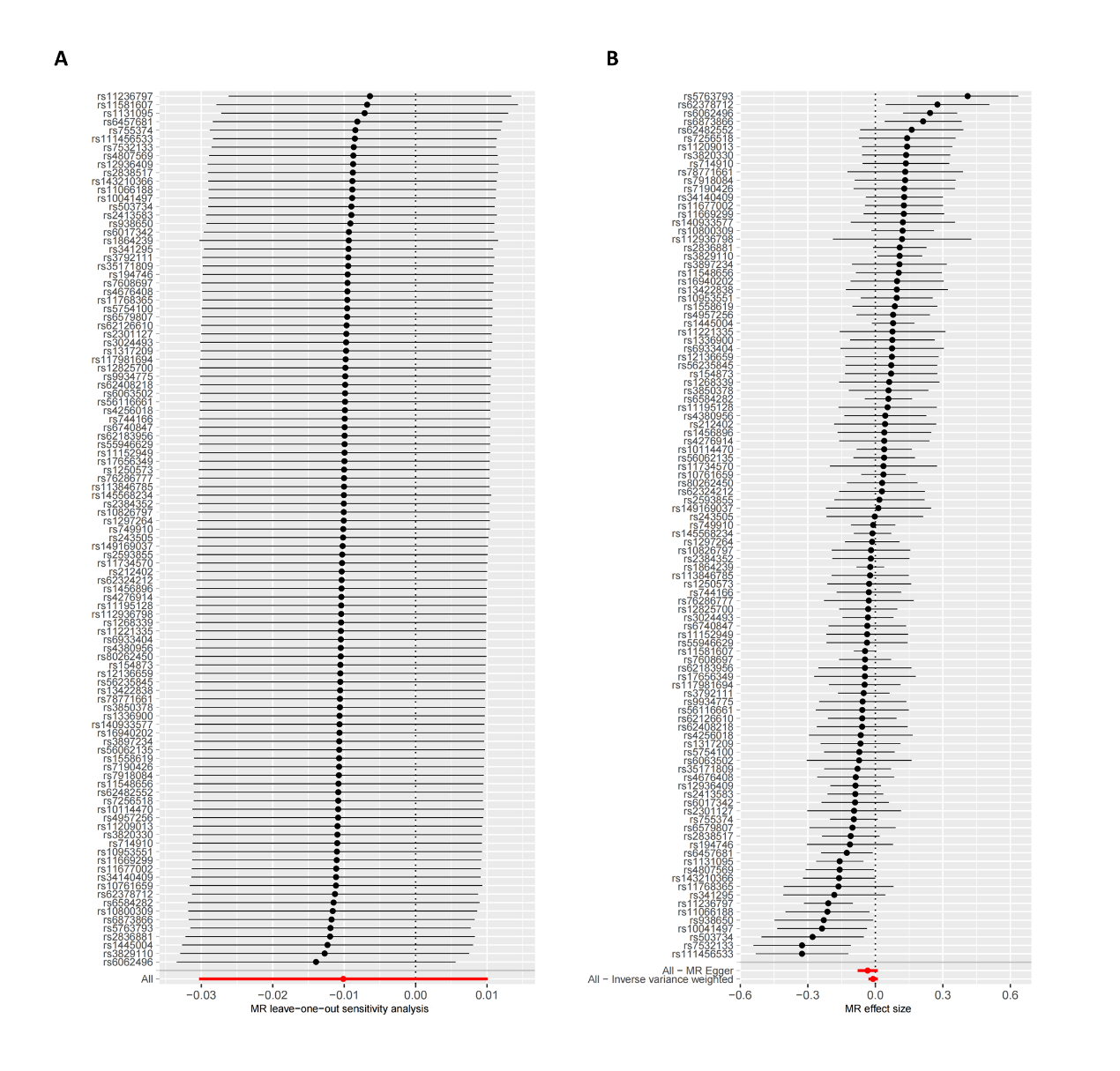


**(A)** The leave-one-out sensitivity analysis plot of the causal effect of IBD on PCa risk. After removing each SNP, the overall error lines did not change much, indicating that the results were reliable. All: the overall effect without removing SNPs. **(B)** The forest plot of the causal effect of IBD on PCa risk. The effect of each SNP was calculated separately, and the overall effect was calculated using MR Egger and IVW methods.

**Supplementary Figure S2**.
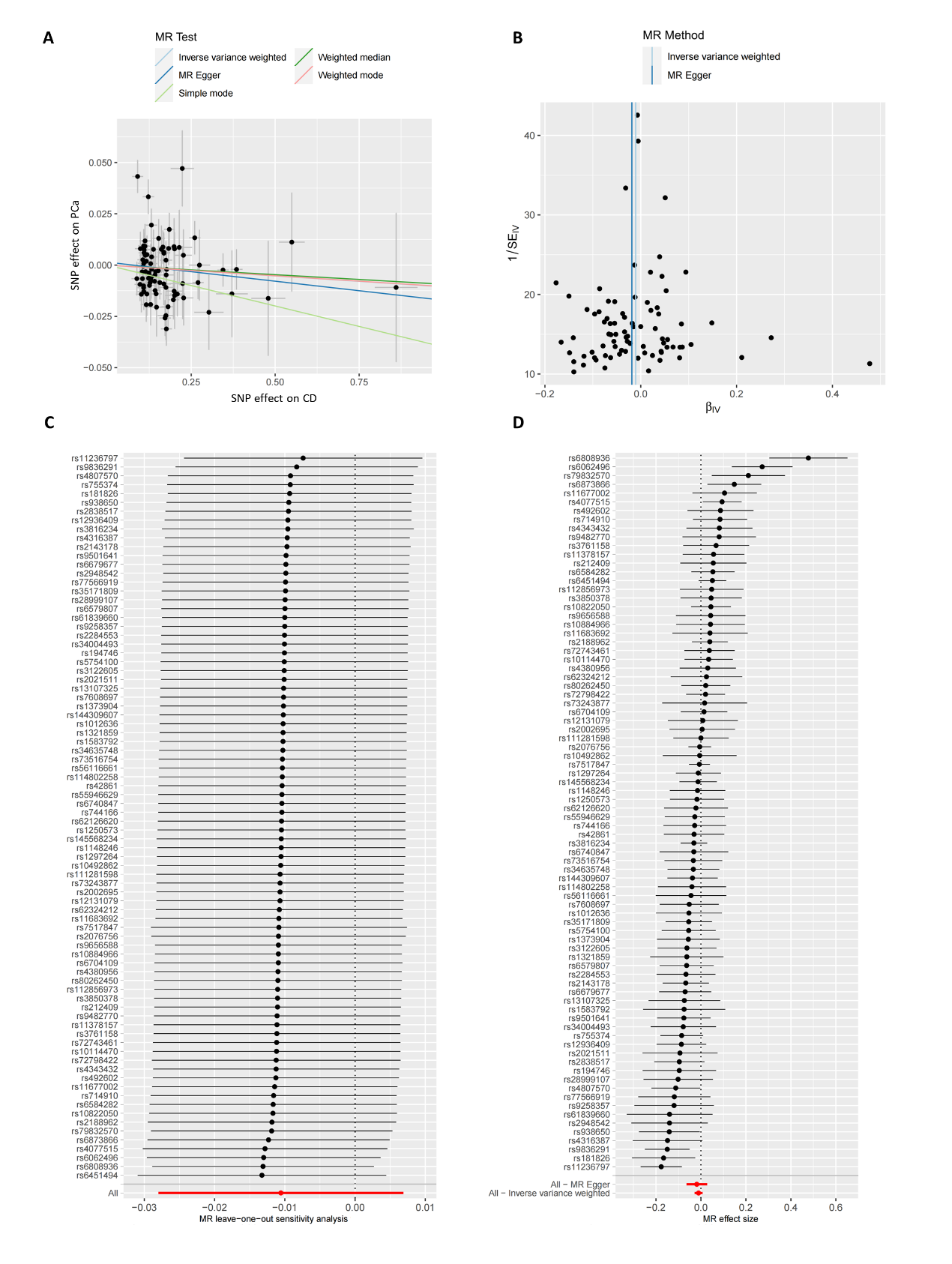


**(A)** The scatter plot of the causal effect of CD on PCa risk. Analyses were conducted using the inverse-variance weighted, MR-Egger, Weighted Median, Simple Mode, and Weighted Mode methods. The slope of each line corresponding to the causal estimates for each method. **(B)** The funnel plot of the causal effect of CD on PCa risk. Individual SNP was delineated in the background. **(C)** The leave-one-out sensitivity analysis plot of the causal effect of CD on PCa risk. After removing each SNP, the overall error lines did not change much, indicating that the results were reliable. All: the overall effect without removing SNPs. **(D)** The forest plot of the causal effect of CD on PCa risk. The effect of each SNP was calculated separately, and the overall effect was calculated using MR Egger and IVW methods.

**Supplementary Figure S3**.


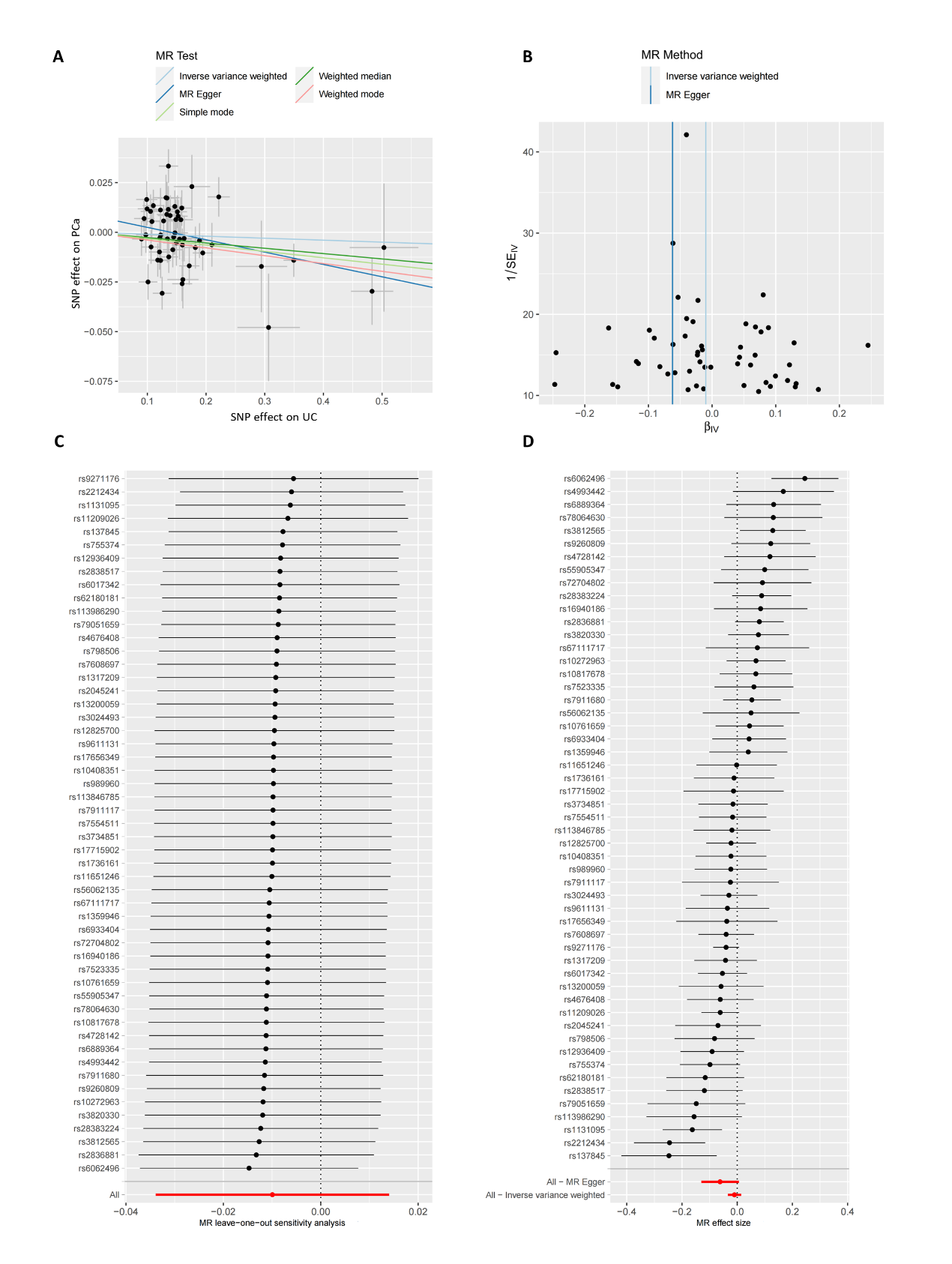


**(A)** The scatter plot of the causal effect of UC on PCa risk. Analyses were conducted using the inverse-variance weighted, MR-Egger, Weighted Median, Simple Mode, and Weighted Mode methods. The slope of each line corresponding to the causal estimates for each method. **(B)** The funnel plot of the causal effect of UC on PCa risk. Individual SNP was delineated in the background. **(C)** The leave-one-out sensitivity analysis plot of the causal effect of UC on PCa risk. After removing each SNP, the overall error lines did not change much, indicating that the results were reliable. All: the overall effect without removing SNPs. **(D)** The forest plot of the causal effect of UC on PCa risk. The effect of each SNP was calculated separately, and the overall effect was calculated using MR Egger and IVW methods.
